# Can RIPASA Scoring System Predict the Pathological Stage of Acute Appendicitis?

**DOI:** 10.1155/2019/8140839

**Published:** 2019-08-01

**Authors:** Banu Karapolat

**Affiliations:** Department of General Surgery, Kanuni Training and Research Hospital, Trabzon, Turkey

## Abstract

**Introduction:**

Being one of the scoring systems used in the diagnosis of acute appendicitis, the RIPASA score can be used easily with a high diagnostic accuracy.

**Objective:**

To evaluate the possible relationship between RIPASA scores and the histopathological examination results of appendectomy materials.

**Materials and Methods:**

This study retrospectively reviews 242 patients who were operated in our clinic between January 2016 and January 2018 with a prediagnosis of acute appendicitis, and the RIPASA scores calculated in the preoperative period were compared to the histopathological examination results of the appendectomy specimens.

**Results:**

The patients consisted of 124 (51.2%) females and 118 (48.8%) males. The ages of the patients ranged from 15 to 81 years. The patients were divided into 3 groups based on their RIPASA scores as low-score (4-7), intermediate-score (7.5-11.5), and high-score (12 and over) groups. There were 20 (52.6%) catarrhal-stage appendicitis cases and 17 (44.7%) normal appendixes in the low-score group; there were 70 (83.3%) catarrhal-stage appendicitis cases, 9 (10.7%) suppurative-stage appendicitis cases, 4 (4.8%) gangrenous-stage appendicitis cases, and 1 (1.2%) perforated appendicitis case in the intermediate-score group. In the high-score group, there were 53 (44.2%) suppurative-stage appendicitis cases, 51 (42.5%) gangrenous-stage appendicitis cases, 11 (9.2%) perforated appendicitis cases, and 5 (4.2%) catarrhal-stage appendicitis cases. A strong positive correlation was found between the RIPASA scores of the patients and the pathological stage of appendicitis (r=0.889; p<0.001).

**Conclusion:**

The RIPASA scoring system can make a correct and prompt diagnosis of acute appendicitis including its possible pathological stage without any need for a computed tomography.

## 1. Introduction

Acute appendicitis is a cause of acute abdomen that mostly requires surgery and is usually diagnosed using clinical history, physical examination findings, and a few laboratory tests [1, 2]. A diagnostic problem arises in approximately 20-33% of the cases in the presence of atypical features and a confusing physical examination and in the absence of typical symptoms and compatible laboratory abnormalities especially at the early stages. Advanced radiological imaging methods such as ultrasonography and computed tomography (CT) are often resorted to for making a quick and accurate diagnosis [3, 4]. Despite all these facilities, the rate of negative or unnecessary appendectomy due to reduced diagnostic accuracy has gone up to approximately 30% [2]. Since these radiological tests are not readily accessible in all medical centers and there was a need to lower this high rate of negative appendectomy, some clinical diagnostic systems based on scoring of various clinical and laboratory findings of patients have been developed for diagnosing of acute appendicitis [3, 4]. One of these, the Raja Isteri Pengiran Anak Saleha appendicitis (RIPASA) score is a useful rapid diagnostic tool used widely across the world and involves 14 clinical parameters (Table 1). The total score ranges from 3 to 16.5; those having a score less than 7 have a low probability of acute appendicitis and those with a score of 7.5 and higher have a high probability of acute appendicitis [5, 6]. With a significantly higher sensitivity, specificity, and diagnostic accuracy, the RIPASA score can help clinicians make the diagnosis of acute appendicitis without any extra tests and carry out the correct management of the patient at an early stage.

This study aimed to compare the RIPASA scores to the histopathological examination results of the appendectomy materials in patients who were operated in our clinic with an initial diagnosis of acute appendicitis to find out if there was any relationship between them and the results obtained were discussed also referring to the literature data.

## 2. Material and Methods

### 2.1. Patients and Study Protocol

In this study, we reviewed retrospectively 242 consecutive patients who presented to the General Surgery Clinic in Trabzon Kanuni Training and Research Hospital between January 2016 and January 2018 due to right iliac fossa pain and who were operated with an initial diagnosis of acute appendicitis. Their RIPASA scores calculated in the preoperative period based on their demographic characteristics such as age and gender, symptoms, physical examination findings, and laboratory results were compared to the histopathological results of their appendectomy specimens. The inclusion criteria were age over 15 years and presenting with right iliac fossa pain. Patients less than 15 years of age and who underwent also an appendectomy during their laparotomy operation performed for some other reason were excluded.

The patients were divided into 3 groups based on their RIPASA scores. The low-score group had scores from 4 to 7, the intermediate-score group had scores from 7.5 to 11.5, and the high-score group had scores 12 and over.

As a result of the histopathological examinations, the appendectomy specimens were reported as normal appendix, chronic appendicitis involving microgranulomatous reaction and reactive lymphoid hyperplasia, catarrhal-stage appendicitis, suppurative-stage appendicitis, gangrenous-stage appendicitis, or perforated appendicitis.

The protocol of this study was approved by the local ethics committee and it was implemented in accordance with the principles of the Helsinki Declaration revised in 2000.

### 2.2. Statistical Analysis

All statistical data analyses were performed using the Statistical Package for Social Sciences (SPSS), version 15.0 for Windows (SPSS Inc., Chicago, IL). Descriptive statistics were used for comparisons. The Chi-Square test was used to compare the distribution ratios of the pathological stages of appendicitis in the RIPASA score groups. The Spearman correlation analysis was performed for the relationship between the RIPASA scores and the pathological stages of appendicitis. The statistical significance level was accepted as p<0.05.

A ROC analysis showed that the cut-off value of the RIPASA score for the diagnosis of acute appendicitis was 6.25 (with 99.6% sensitivity, 88.2% specificity, and a likelihood ratio of 8.4).

## 3. Results

Of the 242 patients in this study, 124 (51.2%) were female and 118 (48.8%) male. The ages of the patients ranged between 15 and 81 with a median: 32 (IQR: 22.7-44).

RIPASA scores of the patients were distributed as follows. The low-score group consisted of 38 (15.7%) patients, the intermediate-score group consisted of 84 (34.7%) patients, and the high-score group consisted of 120 (49.6%) patients.

A review of the pathology results of the patients revealed that 20 (52.6%) patients in the low-score group had catarrhal-stage appendicitis, 17 (44.7%) normal appendix, and 1 (2.6%) chronic appendicitis involving microgranulomatous reaction and reactive lymphoid hyperplasia. In the intermediate-score group, 70 (83.3%) patients had catarrhal-stage appendicitis, 9 (10.7%) patients suppurative-stage appendicitis, 4 (4.8%) patients gangrenous-stage appendicitis, and 1 (1.2%) patient perforated appendicitis. In the high-score group, there were 53 (44.2%) suppurative-stage appendicitis cases, 51 (42.5%) gangrenous-stage appendicitis cases, 11 (9.2%) perforated appendicitis cases, and 5 (4.2%) catarrhal-stage appendicitis cases. A statistically significant difference was found between these 3 groups in the comparison of the pathological stage distribution of their appendicitis specimens (p<0.001).

**Figure 1:**
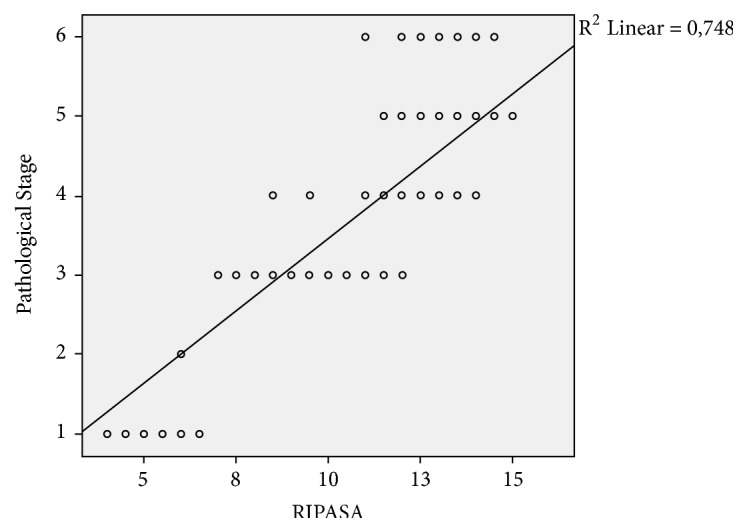
A strong positive correlation is seen between the RIPASA scores and the pathological stage of appendicitis (pathological stages of appendicitis, 1: normal appendix, 2: chronic appendicitis involving microgranulomatous reaction and reactive lymphoid hyperplasia, 3: catarrhal appendicitis, 4: suppurative appendicitis, 5: gangrenous appendicitis, and 6: perforated appendicitis).

A strong positive correlation was found between the RIPASA scores of the patients and the pathological stage of their appendicitis (r=0.889; p<0.001) (Figure 1).

## 4. Discussion

This study underlines five points: (1) In the group with RIPASA scores between 4 and 7, approximately half of the patients had catarrhal-stage appendicitis and the other half normal appendix, (2) in the group with RIPASA scores between 7.5 and 11.5, the majority of the patients had catarrhal-stage appendicitis, (3) most of the patients in this study consisted of those who had a RIPASA score of 12 and greater, and a large majority of these patients had either suppurative- or gangrenous-stage appendicitis, (4) as the RIPASA scores increased, the pathological stages of the patients advanced, and (5) the cut-off value of the RIPASA score for the diagnosis of acute appendicitis was 6.25.

In acute appendicitis, late or incorrect diagnosis leads to aggravation of the existing inflammation, resulting in serious complications including appendicular perforation, peritonitis, intraabdominal abscess, and sepsis, with an increase in morbidity and mortality. The diagnosis of acute appendicitis may be difficult in children and teenagers due to atypical clinical features and in the elderly and females of reproductive age due to a wide range of differential diagnoses [7]. In such cases, use of advanced radiological examinations such as CT may become necessary. However, sometimes the diagnosis of acute appendicitis can only be made based on the intraoperative macroscopic appearance of the appendix tissue and the histopathological examination of the removed appendectomy material [4]. Since these problems have been experienced for a long time in almost all surgical clinics worldwide, many scoring systems have been developed for the diagnosis of acute appendicitis including Alvarado, modified Alvarado, appendicitis inflammatory response score, Ohmann score, and Lintula score. However, because all these scoring systems produced different results in different ethnic groups and had rather low sensitivities and specificities, a need arose to devise new systems. Developed in 2010 and started to be used widely thereafter, the RIPASA score is an inexpensive, easy to use and highly reliable quantitative scoring system that enables to make a correct and early diagnosis of acute appendicitis and to significantly reduce the negative appendectomy rate. In this study, approximately half of the patients in the group with RIPASA scores between 4 and 7 had catarrhal-stage appendicitis and the other half normal appendix. In patients with a RIPASA score less than 7, there is no or very little probability of having an acute appendicitis. Here, the entire patients who were diagnosed with catarrhal-stage appendicitis had a RIPASA score of 7 and they consisted of patients whose ultrasonography showed a normal or invisible appendix but whose physical examination findings were suspicious. These patients were hospitalized and observed for some time and then administered appendectomy as their symptoms showed no improvement.

In our study, most of the patients with a RIPASA score between 7.5 and 11.5 were diagnosed with catarrhal-stage appendicitis. The rest of the patients in this group had appendicitis of a more advanced pathological stage. The patients with a RIPASA score 12 and over comprised the majority of the study population and most of these patients had suppurative- or gangrenous-stage appendicitis. Only 4.2% of the patients in this group had catarrhal-stage appendicitis. As the score increased the number of patients at the catarrhal-stage declined sharply and histopathologically more advanced stages, primarily suppurative- and gangrenous-stages, became predominant. No CT scan was performed in any of these patients in the intermediate- and high-score groups. With 94% sensitivity and 95% specificity, CT has actually been used widely for many years in making a definite diagnosis of acute appendicitis [8]. However, unavailability of CT in every health centre and the delay caused during the scan may pose problems for emergency appendectomy, increasing the risk of further appendicitis-related complications. CT is also an expensive procedure raising the cost of healthcare and patients are exposed to radiation. There are studies in the literature reporting that unnecessary CT scans lead to unnecessary appendectomies in patients with early low-grade appendicitis which can be resolved spontaneously with antibiotics therapy and this means that such patients are burdened with surgical risks [8]. Appendicitis was found in the histopathological examinations of the entire patients in these intermediate- and high-score groups who had RIPASA scores higher than 7.5. When this is taken into consideration together with the above-mentioned disadvantages of CT, we think a CT scan is not necessary for patients with a RIPASA score 7.5 and over. This type of a practice will fully justify the validity of the existence of a RIPASA scoring system. In this context, it will be useful to inform physicians, particularly those working in rural hospitals without a CT unit, about the necessity to use the RIPASA scoring system more frequently.

It was observed in this study that as the RIPASA scores increased, the pathological stages of appendicitis advanced and nearly all patients with a high score had suppurative, gangrenous, or perforated appendicitis. When the severity of inflammation increases in the appendix tissue, the clinical and laboratory findings are influenced and high scores are obtained in the RIPASA scoring system where these data are used systematically. This information combined with the strong positive correlation found between the RIPASA scores and the pathological stage of appendicitis suggests that the pathological stage of appendicitis can be predicted using the RIPASA scores. In this way, when it comes to a patient with a high score, the surgical team will be able to both decide on operation more easily and take the necessary measures in advance knowing that they may encounter a more complicated appendicitis during the operation.

The cut-off value of the RIPASA score for the diagnosis of acute appendicitis was found to be 6.25 in this study. This score is lower than the conventional RIPASA cut-off value of 7.5. This may be thought of as a local result associated with the characteristics of the Turkish population [9, 10].

The limitations of this study include its retrospective character, having been conducted in a single centre, and small number of patients. Our results may gain more value with prospective multicentre studies including larger patient populations to be carried out in the future.

In conclusion, making a correct and prompt diagnosis of acute appendicitis including its possible pathological stage is possible with the RIPASA score, which is easily obtained using simple clinical and laboratory data, without a need for CT.

## Figures and Tables

**(a):** 

Patients characteristics	Score

Female	0.5

Male	1

Age <40 years	1

Age >40 years	0.5

Right iliac fossa pain	0.5

Pain migration to right iliac fossa	0.5

Anorexia	1

Nausea and vomiting	1

Duration of symptoms <48 hours	1

Duration of symptoms >48 hours	0.5

Right iliac fossa tenderness	1

Guarding	2

Rebound tenderness	1

Rovsing's sign	2

Fever	1

Raised WCC	1

Negative urinalysis	1

Total	16.5

**(b):** 

RIPASA score	Diagnosis of acute appendicitis

5.0 >	Acute appendicitis is not possible

5-7.0	Low probability of acute appendicitis

7.5- 11.5	High probability of acute appendicitis

12 <	Absolutely acute appendicitis

## Data Availability

The data used to support the findings of this study are available from the corresponding author upon request.
